# Bioactive Coatings for Orthopaedic Implants—Recent Trends in Development of Implant Coatings

**DOI:** 10.3390/ijms150711878

**Published:** 2014-07-04

**Authors:** Bill G. X. Zhang, Damian E. Myers, Gordon G. Wallace, Milan Brandt, Peter F. M. Choong

**Affiliations:** 1 Departments of Surgery, University of Melbourne, St Vincent’s Hospital (Melbourne), Fitzroy, VIC 3065, Australia; E-Mails: billzhang11@gmail.com (B.G.X.Z.); damianem@unimelb.edu.au (D.E.M.); 2 Department of Orthopaedics, St Vincent’s Hospital (Melbourne), Fitzroy, VIC 3065, Australia; 3 Australian Research Council Centre of Excellence for Electromaterials Science (ACES), University of Wollongong, Intelligent Polymer Research Institute, Wollongong, NSW 2500, Australia; E-Mail: gwallace@uow.edu.au; 4 School of Aerospace, Mechanical and Manufacturing Engineering, RMIT University, Bundoora, VIC 3083, Australia; E-Mail: milan.brandt@rmit.edu.au

**Keywords:** osteointegration, orthopaedic, implant, coating, osteoinduction, osteoconduction, infection, antimicrobial, silver, biofilm

## Abstract

Joint replacement is a major orthopaedic procedure used to treat joint osteoarthritis. Aseptic loosening and infection are the two most significant causes of prosthetic implant failure. The ideal implant should be able to promote osteointegration, deter bacterial adhesion and minimize prosthetic infection. Recent developments in material science and cell biology have seen the development of new orthopaedic implant coatings to address these issues. Coatings consisting of bioceramics, extracellular matrix proteins, biological peptides or growth factors impart bioactivity and biocompatibility to the metallic surface of conventional orthopaedic prosthesis that promote bone ingrowth and differentiation of stem cells into osteoblasts leading to enhanced osteointegration of the implant. Furthermore, coatings such as silver, nitric oxide, antibiotics, antiseptics and antimicrobial peptides with anti-microbial properties have also been developed, which show promise in reducing bacterial adhesion and prosthetic infections. This review summarizes some of the recent developments in coatings for orthopaedic implants.

## 1. Introduction

Joint arthroplasty (replacement) is a surgical procedure whereby the patient’s joint is replaced by an implant. It is one of the most frequently performed procedures for the treatment of end-staged joint degeneration (osteoarthritis), which is characterised by pain, loss of joint function and deformity. With an aging population, the global burden of disease associated with osteoarthritis is expected to rise, increasing future demand for this procedure. Currently, almost 100,000 joint replacements are performed in Australia each year, mostly for osteoarthritis. Between 8.3% and 12.1% of these are revision arthroplasties performed for implant failure mainly due to aseptic loosening (28%–29%) and implant infection (15%–20%) [1]. Aseptic loosening occurs secondary to debris particles arising from wear products at the articulating surfaces or from cement disintegration at the cement-bone or cement prosthesis interfaces after long periods of repetitive mechanical stress associated with locomotion. These wear particles lead to a biologic response characterised by an inflammatory response in the immediately adjacent bone that culminates in bone loss and loosening of the implant. The incidence of aseptic loosening of joint prosthesis 10 years after surgery is approximately 2% for knee and hip replacements [1]. 

Where no cement is used (cementless arthroplasty), one of the key determinants of risk of loosening without infection (aseptic loosening) is the degree of “osteointegration” of the prosthesis into the bone. Osteointegration refers to the process whereby bone grows directly onto or into the implant surfaces [2]. Currently, most implants are made of metals such as cobalt chrome alloy, stainless steel or titanium alloy. However these metals generally lack a biologically active surface that either encourages osteointegration or wards off infection. Attention has thus been focused on developing various coatings to supplement the function of current implants [3,4,5,6,7,8,9,10]. The design of these coatings must satisfy several important criteria: firstly the coating must be biocompatible and not trigger significant immune or foreign-body response; secondly, it must be “osteoconductive” in its promotion of osteoblasts (cells that make bone) to adhere to, proliferate and grow on the surface of the implant to form a secure bone-implant bonding; thirdly, the implant must also be “osteoinductive” and be able to recruit various stem cells from surrounding tissue and circulation and induce differentiation into osteogenic cells [2]. Furthermore the coating must have sufficient mechanical stability when under physiological stresses associated with locomotion to not detach from the implant surface; Finally, the implant coating should have anti-microbial properties minimizing the risk of prosthetic infection. Currently none of the commercially available prosthesis are able to satisfy all of the above criteria, further emphasizing the need for research and development of new biological coatings for orthopaedic implants.

Convergence and improvements in manufacturing, cell biology and material science have led to development of novel biological coatings with osteoconductive as well as osteoinductive properties that emulate the natural niche of growing bones. Micro and nano-structured coatings functionalized with bioceramics and osteogenic bioactive molecules and drugs have been shown to accelerate osteointegration of implants in various *in vitro* and *in vivo* experimental models [3,4,5,6,7,8,9,10]. In addition, there has been ongoing research to develop anti-infective surface coatings using silver (Ag^+^) ions, nitric oxide (NO), antibiotics and antimicrobial peptides to inhibit bacterial infection to dissipate the risk of prosthetic infection [11,12,13,14,15,16,17,18]. The aim of this review is to discuss recent approaches towards improving the integration of orthopaedic prosthesis through novel implant coatings. The first section of this review explores recent trends in coatings that promote osteointegration. The effect of coating surface topography on osteogenic cells is summarized followed by an outline of the use of various calcium phosphate ceramics, extracellular matrix molecules (ECM), bioactive peptides and growth factors that are complexed to orthopaedic implants to enhance bony ingrowth. The second part of this review summarizes developments in new anti-infective orthopaedic coatings.

## 2. Cell Response to Surface Features of Implant Coatings

In order to design the ideal coating for orthopaedic implants, the response of osteogenic cells to micro- and nano-scale architecture surfaces must first be elucidated. Much research has focused on examining the effect of surface architecture on osteogenic cell differentiation and adhesion. In the following paragraphs the effect of surface roughness, microtopography, nanotopography, porosity and surface energy on osteogenic cell function and osteointegration will be examined. 

### 2.1. Surface Roughness and Microtopography

Surface roughness affects both osteoblast adhesion and differentiation. Osteoblast-like cells grown on rough titanium surfaces (Ra 4–7 μm) show reduced proliferation and enhanced osteogenic differentiation with up-regulation of alkaline phosphatase (ALP) activity and the osteogenic differentiation marker osteocalcin [19,20,21,22,23,24,25]. This differentiation effect of rough surfaces is likely mediated by integrin α2β1 with upregulation of a range of osteogenic growth factors including Transforming Growth Factor 1 (TGF-1), Prostaglandin E_2_ (PGE_2_), Wnt pathway agonist Dickkopf-related protein 2 (Dkk 2), Vascular Endothelial Growth Factor (VEGF), Epidermal Growth Factor (EGF) and Fibroblast Growth Factor (FGF) [25,26,27,28]. VEGF is an angiogenic factor while EGF, FGF and TGF-1 are potent mitogenic factors for osteoblasts and mesenchymal stem cells [29]. Both Dkk 2 and PGE_2_ promote differentiation of osteoblasts [30,31]. PGE_2_ is instrumental in roughness-induced cell differentiation. Inhibition of PGE_2_ production by indomethacin blocked expression of osteogenic differentiation markers in cells grown on rough surfaces [19,21]. In addition to their effect on osteoblasts, micro-rough surfaces (Ra 4–5 μm) also inhibit osteoclast (cells that remove bone) activity by upregulating receptor activator of nuclear factor kappa-B ligand (RANKL) and decoy receptor osteoprotegerin (OPG) on osteoblasts. Binding of RANKL by OPG prevents it from binding and activating osteoclasts through the RANK receptor, thus indirectly promoting net bone deposition [24,32]. Currently, various implants used in clinical practice contain surface micro-pits and depressions. The surface features can be engineered through techniques such as grit-blasting, acid etching and plasma spraying [33]. These micro-textured implants show enhanced osteointegration compared to smooth implants when implanted *in vivo* into bone [34].

### 2.2. Nanotopography

**Figure 1:**
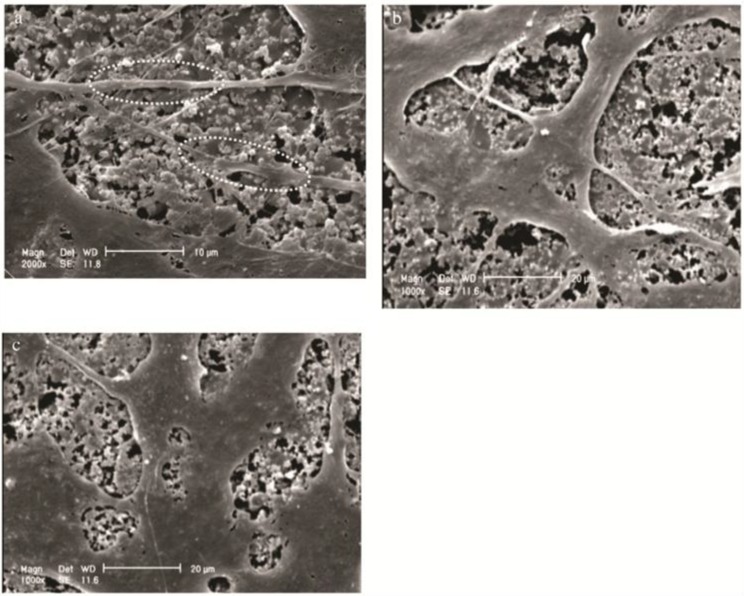
Bone cells show enhanced spreading and extension of filopodium (white dotted ovals) when cultured on nanostructured surfaces. SEM (scanning electron microscopy) images of ROS17/2.8 cells grown on nanostructured HA/TiO_2_ substrates for (**a**) 3 (**b**) 6 and (**c**) 9 days. Note the increased cell spreading over time with filopodial extension. Reprinted from [35] with permission from Elsevier, Copyright 2014.

Much of the natural environment surrounding osteoblasts and osteoclasts consist of structures with nano-scale topography. Collagen fibrils and HA (hydroxyapatite) crystals have lengths ranging from 50 to 300 nm and width of 0.5–5 nm [33]. As a result, metal surfaces with nano-scale architecture have been devised in an attempt to recapitulate the physiological environment of growing bone. Nanoscale architecture is defined by feature or grain size less than 100 nm. This architecture affects roughness, surface area and surface energy of the material and can thus enhance osteoblast contact signalling. Nanophase titanium surfaces with grain size <100 nm, have been shown to be more effective in promoting osteoblast adhesion and proliferation compared to microtextured surfaces (grain size > 100 nm) [35,36,37,38,39]. Upon adhering to the nanotextured surface, osteoblasts show enhanced cell spreading and filopodial extension [37] (Figure 1). 

**Figure 2:**
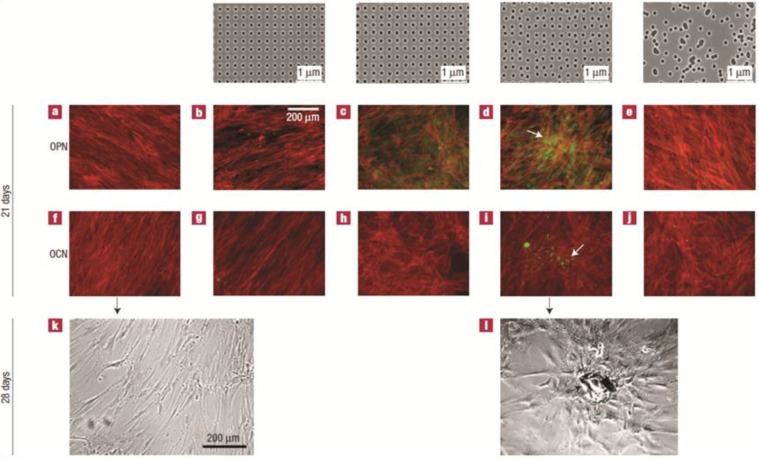
Mesenchymal stem cells (MSC) are sensitive to nanotopography and show enhanced osteogenic differentiation when cultured on surfaces with semi-ordered architecture. MSCs are cultured on planar PMMA (poly-methyl methacrylate) (**a**,**f**,**k**), PMMA surfaces with pits (120-nm-diameter and 100 nm deep) arranged in square arrays (300 nm apart) (**a**,**g**), PMMA surfaces with pits displaced +/−20 nm from perfect square arrangement (**c**,**h**), PMMA surfaces with pits displaced by +/−50 nm (**d**,**i**,**l**) and PMMA with completely randomly patterned pits. Top row show the nanotopography of the different PMMA surfaces. (**a**–**e**) Cells co-stained with alizarin-red and antibodies against osteopontin (OPN); (**f**–**j**) Cells co-stained with alizarin-red and antibodies against osteocalcin (OCN); (**k**–**j**) Phase contrast microscope image of MSCs grown on planar (**k**) and semi-ordered (**J**) PMMA surfaces. Note that MSCs grown on pits displaced by 20 and 50 nm show enhanced osteogenic differentiation and raised OCN and OPN with nodules forming in cells on 50 nm displaced surfaces (arrow in **d** and **i**). Cells grown on planar surfaces and surfaces with pits in ordered array show no osteogenic differentiation and maintain fibroblast morphology (**a**,**b**,**f**,**j**,**k**). This contrasts with bone nodules forming on cells grown on surfaces with pits displaced by 50 nm (arrow) (**l**). Reprinted from [47] with permission from Macmillan Publishers Ltd, copyright 2014.

The underlying mechanism of the enhanced adhesion is likely related to the increased protein adsorption on nanoscale surfaces. Binding of proteins such as vitronectin to the nanophase surface induces conformational change on vitronectin exposing more cell binding sites for anchoring osteoblasts [38,40,41,42]. In addition to promoting adhesion, nanotopography can enhance osteogenic differentiation in osteoblasts [40,42,43,44] and affect osteoclast activity. Osteoclast-like cells grown on nanophase alumina (grain size < 100 nm) show increased tartrate-resistant acid phosphatase (TRAP) activity and resorption pits on the substrate, indicative of increased bone resorption [45]. The cellular response to nanotopography varies according to the level of differentiation of the cell. Undifferentiated mesenchymal stem cells do not show osteogenic differentiation in response to nano-scale topography while osteoblasts show enhanced differentiation when grown on the same surface [46]. In addition to general scale of architecture the way that the various nanoscale structures are arranged on the surface can also affect both osteoconduction and osteoinduction. Mesenchymal stem cells grown on poly-methyl methacrylate (PMMA) substrates that have semi-ordered nanoscale surface pit arrangement show superior differentiation and TGF β1 expression compared to cells grown on surfaces with pits organized in perfect hexagonal or square arrays [47] (Figure 2).

### 2.3. Porosity

Surface porosity impacts on osteointegration by allowing direct ingrowth of osteogenic cells into the implant, thereby strengthening the bone-implant interface [48]. A number of research groups have investigated the effect of pore morphology and dimension on osteoblast differentiation and osteointegration. It is generally agreed that scaffolds with interconnected pores show enhanced bony ingrowth compared with those with closed pores [46]. This is attributed to improved ingrowth of vasculature resulting in better delivery of osteoprogenitors to the scaffold bulk [49]. Furthermore, it has been proposed that pores must be sufficiently large for vascular infiltration without compromising the mechanical properties of the coating and that an optimal pore size exists. This is supported by observations that pore sizes greater than 1mm promote fibrotic tissue ingrowth in preference to bone, which is not ideal [48]. Studies along these lines concur that ideal pore size lies within a range between 100 and 700 μm depending on the morphology of the pores, the composition of the scaffolds and the manufacturing technique [50,51,52,53,54,55]. 

### 2.4. Surface Energy

Surface energy, also known as surface wettability, enhances both osteoblast adhesion and differentiation. Osteoblasts grown on high surface energy (hydrophilic) substrates display increased cell adhesion, proliferation and upregulation of various differentiation markers such as osteocalcin, type-I-collagen, osteoprotegerin, and glyceraldehyde-3-phosphate-dehydrogenase and raised ALP activity [56,57,58]. This cell adhesion is likely mediated by integrin α5β3 and increased adhesion related molecule focal adhesion kinase (FAK) [57,59]. In addition, osteoblasts grown on hydrophilic surfaces also secrete osteogenic factors such as PGE_2_ and TGF β1 [43]. Surface energy has also been shown to influence mesenchymal cell differentiation. Hydrophilic surfaces influenced stem cell differentiation into osteogenic cells and bolstered bone mineral deposition [60]. The surface energy of metals can be improved by incorporating various charged functional groups to the surface with encouraging results in both *in vitro* and *in vivo* studies [61,62,63]. These functionalization methods will be discussed in more detail later in the section on “metal surface functionalization and ion incorporation”. 

## 3. Implant Surface Enhancements for Enhanced Osteointegration

A range of biologically active materials have been studied as potential coatings for orthopaedic implants. These can be grouped broadly into calcium phosphate-based bioceramics, metal ion incorporated coatings, ECM components and peptides, titanium nanotubes and coatings that act as sustained delivery devices for osteogenic growth factors and drugs.

### 3.1. Calcium Phosphates

**Figure 3:**
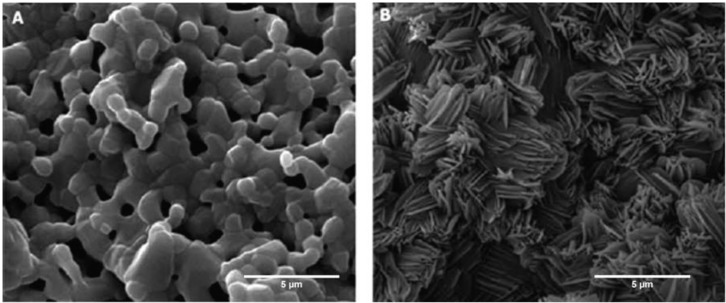
Calcium phosphate based ceramics attract natural apatite deposition on its surface after immersion in physiological solutions. This occurs through an ion exchange reaction between the calcium phosphate in the ceramic coating and the ions and proteins in the surrounding solution. SEM images of BCP (bi-phasic calcium phosphates) scaffolds before and after immersion in phosphate buffered saline (PBS). (**A**) HA/TCP scaffolds before immersion in PBS; (**B**) HA/TCP scaffolds after immersion in PBS for 2 weeks. Note the deposition of apatite crystals on the scaffold surface. Reprinted from [82] with permission from Bentham Open, copyright 2010.

Calcium phosphates form an integral part of natural apatite bone minerals. Various forms of calcium phosphate have been examined as coatings for orthopaedic prostheses. In this group of materials, the most thoroughly researched and characterized calcium phosphate is hydroxyapatite (HA). Hydroxyapatite is an osteoconductive material that has been shown, in both *in vitro* and *in vivo* models, to promote osteoblast adhesion and in some studies differentiation [6,64,65,66,67,68,69,70]. Furthermore, HA coatings have been studied in a large body of clinical trials in humans [71,72,73,74,75,76,77,78,79,80,81]. Like all other calcium phosphates, HA induces a layer of carbonate-hydroxyapatite to form on its surface soon after it is implanted *in vivo* [82,83,84,85]. This is a result of an ion exchange process with the environment whereby calcium and phosphate ions are released from the implant while proteins from the physiological solution are simultaneously deposited onto the HA (Figure 3). The resulting coating layer on the HA is known as carbonate-hydroxyapatite (CO-HA) and it resembles the apatite present in normal bone [86]. Compared to HA, CO-HA also contains CO_3_, HPO_4_, F, Cl, Mg, Na, K ions, and some trace elements (such as Sr and Zn) [87]. The new apatite layer acts as a scaffold for osteoblasts and is further resorbed by osteoclasts over time and replaced by new bony tissue [84]. One of the main drawbacks of HA is its brittle nature and poor mechanical properties [88]. As a result, it is often used as a biologically active coating for metal prosthesis. 

HA-coated implants have been examined in many clinical trials of arthroplasties with disparate results. Some studies show improvements in osteointegration of implants coated with HA [71,72,73,89] while other studies fail to show any benefit [74,75,76,78,79,80,81]. The disparity in results likely stems from various surgeon and patient factors that often confound clinical trials. One mechanism of failure of HA-coated implants revealed by the studies involves delamination and resorption of the HA coating due to poor implant-coating attachment [90,91]. Loss of HA coating leads to micromotion of the implant and increased fretting and production of debris particles [92]. As a result new techniques of coating implants with HA have been developed. These include plasma spraying, thermal spraying, sputter coating, pulsed laser deposition, dip coating, sol-gel, electrophoretic deposition, hot isostatic pressing and ion-beam assisted deposition [90]. For a detailed review of these techniques the reader is referred to an excellent review by Mohseni *et al.* [90], but it should be noted that these techniques lead to differential surface effects that compound cellular response to the material composition *per se*. Despite inconsistent results in clinical trials, perhaps due to such differential effects, HA coatings have delivered improved osteointegration in multiple *in vivo* animal studies. HA-coated titanium implants inserted into the femur of dogs promoted increased bony ingrowth at 6 weeks after surgery compared to uncoated titanium implants. This contrasts with the fibrotic tissue that develops between the bone and uncoated implants [6,69].

The amount of CO-HA that forms on calcium-based bioceramic coatings is determined by the amount of soluble calcium phosphate in the coating. Calcium phosphate ceramics exists in many forms or “phases”. HA is relatively insoluble calcium ceramic while tri-calcium phosphate (TCP) is a more soluble counterpart. Coatings consisting of a combination of HA and TCP are known as bi-phasic calcium phosphates (BCP). The TCP in the BCP readily dissolves in the body releasing more ions, increasing the amount of carbonatehydroxyapatite that forms on the surface [82,83,93]. BCP containing scaffolds are both osteoconductive and osteoinductive, promoting osteogenic differentiation of mesenchymal stem cells (MSC) and bone formation in extra-skeletal sites in various animal models [7,94,95,96,97,98]. However, one must be cautious before translating these results into human applications as there is a high degree of interspecies variability in the capacity of different animals to form ectopic ossification in non-skeletal sites [98,99]. More standardization of animal models of ectopic ossification is required to clarify and consolidate the existing data from published studies in this field. In addition to TCP many other soluble calcium phosphate compounds have also been investigated as osteogenic scaffolds including monocalcium phosphate monohydrate (MCPM), monocalcium phosphate anhydrous (MCPA or MCP) and dicalcium phosphate dihydrate (DCPD) [93]. Amongst these compounds, DCPD, also known as brushite, has been used as a coating on commercially available hip and ankle replacement prosthesis with encouraging results in clinical trials [100,101]. Brushite is more soluble than TCP potentially allowing for increased apatite formation when exposed to physiological fluids [93,102,103,104]. Furthermore brushite can be deposited more homogenously on irregularly shaped prosthesis [105]. Human osteoblasts grown on brushite coatings show enhanced differentiation and ECM production compared to non-coated titanium surfaces [106]. Titanium implants with brushite coatings enhanced bone ingrowth when implanted into rabbit femurs [105]. However more *in vivo* studies are needed to compare the performance of brushite coating with other forms of calcium phosphate coatings. 

The physical morphology and chemical composition of calcium phosphate ceramics can be adjusted to maximize osteoinductive potential. Both porosity and the ratio of TCP to HA have been shown to affect the amount of bone formed on the scaffolds in extra-skeletal sites [107]. Porous calcium phosphates with increased micropores (pores < 10 μm) are more osteoinductive than their non-porous counterparts. The optimal pore size must lie within an optimal range between 100 and 500 μm to be large enough to allow vascular infiltration and small enough to not impact on mechanical properties [54,55,108,109]. The TCP content of BCP also affects osteoinduction. BCPs with higher TCP content are more osteoinductive than those with higher HA content [96,110]. TCP likely imparts a twofold advantage on bone formation. Firstly, as mentioned earlier, TCP promotes natural apatite deposition therefore acting as a bioactive interposing layer between the coating and new bone. Secondly, TCP introduces pores to the scaffold as it rapidly dissolves. The content of TCP also influences scaffold performance. When the TCP content is too high, the structural integrity of the scaffold is compromised as the excessively porous scaffold collapses, losing its porous architecture in the process [110]. Clearly there needs to be a balance between the ability of the implant coating to exchange ions with the environment and the maintenance of structural integrity to allow sufficient time for bony ingrowth. The exact mechanisms involved in BCP stimulated osteoinduction and the role of natural apatite deposition in bone ingrowth is unclear. Both calcium and osteoclast activity have been implicated as mediators of calcium phosphate induced osteoinduction [111].

### 3.2. Metal Surface Functionalization and Ion Incorporation

**Figure 4:**
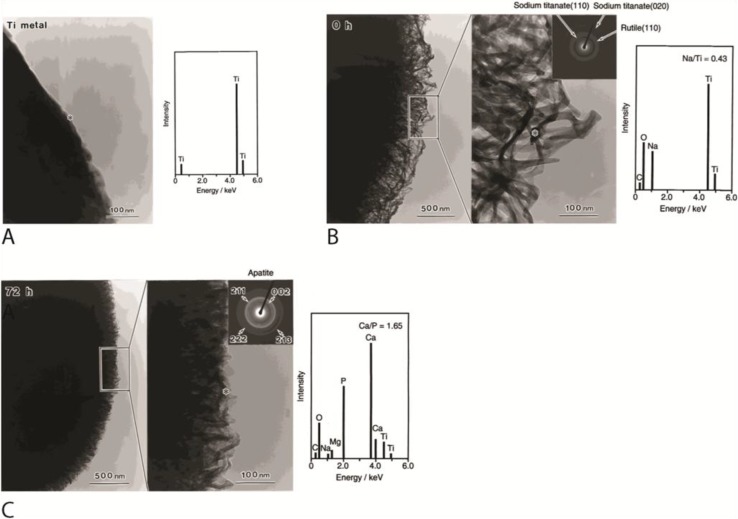
Functionalization of Titanium surfaces with hydroxyl (OH^−^) groups enhanced nucleation of bone like apatite on the metal surface when it is submerged in physiological solutions. The figure shows transmission electron microscopy (TEM) images and energy dispersive X-ray spectrometry (EDX) results of titanium surfaces treated with NaOH and heat followed by immersion in physiological solution. (**A**) Titanium surface before NaOH and heat treatment; (**B**) Titanium surface after NaOH and heat treatment. Note the layer of amorphous sodium titanate that forms on the titanium surface; and (**C**) NaOH and heat-treated titanium surface after 72 h immersion in physiological solution. Note the deposition of natural apatite on the implant surface. * Center of electron diffraction and EDX analysis. Reprinted from [62] with permission from John Wiley & Sons, Inc., Copyright 2014.

Unprocessed metal implants usually possess bio-inert hydrophobic surfaces. This can be overcome by functionalizing the metal surface with reactive hydroxyl groups (OH) to impart a hydrophilic surface. The functionalization process can be accomplished by various techniques such as NaOH treatment and submersion in ionic solutions under conditions that are isolated from the atmosphere [43,63,112,113,114]. The functionalized implants generally promote nucleation of natural apatite crystals and adsorption of ECM molecules, such as fibronectin, to the implant surface when it is submerged in physiological solutions [61,62,115] (Figure 4). 

Metal implants with hydroxylated surfaces promote osteointegration *in vivo* and bone formation when implanted in extra-skeletal sites [43,63,114]. More recently, metals incorporated with calcium, phosphorous, magnesium and fluoride ions also show promising results in promoting osteointegration [5,116,117,118,119,120,121,122,123,124,125,126,127]. Like functionalized metallic implants, these ion incorporated surfaces also promote deposition of natural apatite through an ion exchange reaction [116]. The osteointegrative effects are likely mediated by an increase in osteogenic differentiation of MSC, expression of integrins α1, α2, α5, and β1, and upregulated BMP2 (bone morphogenetic protein 2) secretion by osteoblasts [117,121,125,128]. 

### 3.3. ECM (Extracellular Matrix Molecules) Components and Biological Peptides

Various ECM components have shown potential as materials for improving the performance of orthopaedic implants. Collagen 1 is one of the most studied materials. Collagen 1 is a major component of bone matrix, making up to 80% of the protein in the matrix [129]. Osteoblasts and MSC grown on collagen 1-coated metals show enhanced cell adhesion, mediated through an integrin β1 based pathway [130,131,132]. Collagen 1 coated metallic implants also promote osteointegration and bone-implant apposition *in vivo* [131,133,134]. The effect of collagen 1 coating can be further enhanced by co-immobilization of implants with cartilage ECM molecule sulfated glycosaminoglycan chondroitin sulphate [9,135].

There are some disadvantages associated with use of ECM molecules. Firstly, most ECM molecules are biologically derived and increase the risk of inadvertent introduction of microbes and infectious material into the host during implantation. Secondly biologically-derived molecules often suffer from significant batch-to-batch variability in quality. To overcome these problems various artificial peptides emulating active sequence motifs on the ECM molecules have been developed. One of the most well-known peptides is the Arg-Gly-Asp (RGD) peptide. RGD peptide represents sequences on the 10th type 3 repeat on the main cell binding domain of fibronectin [136], associated with generalised cell adhesion. RGD promotes osteoblast adhesion through an integrin α2β1 pathway [132,137,138]. Apart from fibronectin, RGD is also the active sequence of matrix molecules OPN, bone sialoprotein (BSP) and vitronectin (VN) that promotes osteoblast adhesion [139]. RGD functions mainly as an osteoconductive coating with minimal effects on osteoinduction [140]. RGD-coated titanium implants improve implant osteointegration in various animal studies [10,70,141]. The anchoring of RGD to the implant surface is an important factor affecting osteointegration. RGD peptides that detach from the substrate may inhibit osteoblast adhesion by competing with attached RGD for integrin receptor on osteoblasts [142]. Various methods are available to reliably immobilize RGD to implant surfaces, including direct physical adsorption and chemical immobilization with a spacer molecule and immobilization through an interposing layer of hydroxyapatite [10,143,144].

The aspartic acid residue on RGD peptides predisposes it to *in vivo* degradation. One solution to this problem is to cyclize the molecule to form a cyclic RGD peptide. The increased rigidity imparted by the ring structure of the cyclic peptide minimizes its degradation [145]. Compared to linear RGD, cyclic RGD binds integrins with 20–100 more affinity and shows greater preference for integrins αIIbβ3, αVβ3 and ανβ5 [146]. Titanium implants functionalized with Cyclo-(DfKRG) peptide are more osteoinductive than those with linear RGD and are more able to stimulate peri-implant bone formation *in vivo* [147,148,149].

RGD peptides only emulate one of many bio-active cell binding domains on fibronectin [150,151]. Some of the active motifs on fibronectin can supplement the function of the RGD domain such as the proline-histidine-serine-arginine-asparagine (PHSRN) residue. PHSRN is present on the ninth type 3 repeat on fibronectin [152]. PHSRN bolsters the RGD induced osteoblast spreading and adhesion when it is co-presented with the RGD in a specific spatial array [153,154]. The spatial relationship between RGD and PHSRN must match the relative positions of the two domains on the fibronectin molecule. More recently whole fibronectin fragments (FNIII7-10) containing multiple complementary domains of fibronectin have been synthesized to promote cell adhesion [155]. These FNIII7-10 fragments contain the RGD and PHSRN motifs arranged in the correct spatial relationship. Cells grown on FNIII7-10-containing substrates show superior proliferation, adhesion and focal adhesion kinase (FAK) activation [155]. Unlike RGD, FNIII7-10 possess a greater specificity for integrin α5β1 which is important for differentiation of pre-osteogenic stem cells [155,156]. FNIII7-10 coated titanium implants promote osteogenic differentiation of MSC *in vitro* and osteointegration *in vivo* [156].

Apart from RGD, other peptides that are evaluated as orthopaedic implant coatings include DLTIDDSYWYRI and GFOGER. DLTIDDSYWYRI is an active motif from the large globular 1 domain of human laminin-2 a2 chain that promotes osteoblast differentiation [157,158]. DLTIDDSYWYRI acts through syndecan-1 on the cell membrane resulting in phosphorylation of downstream protein kinase C (PKC) delta leading to cell adhesion and enhanced osteointegration of implants coated with the peptide *in vivo* [158,159]. GFOGER is a peptide which resembles sequences on the collagen I α1(I) chain. It binds α2β1 integrin and promotes cell adhesion [160]. GFOGER coated titanium implants strengthen bone-implant interface bond *in vivo* [161]. More recent studies have combined multiple biological peptides RGD, PHSRN, tyrosine-histidine sequence (YH), and glutamic acid-proline-aspartic acid-isoleucine-methionine (EPDIM) into one coating thus effectively stimulating multiple signalling pathways to promote osteointegration [162].

### 3.4. Titanium Nanotubes

Given the differentiating effects of nanophase architecture on osteoblasts, some researchers have used titania nanotubes as a means of creating nanotextured implant coating. Vertically oriented titania nanotubes enhance osteoblast differentiation and raise osteocalcin expression and integrin/focal contact [163,164]. The behaviour of osteoblasts can be also be regulated by altering the diameter of the nanotubes. Osteoblasts grown on nanotubes with diameter of 30 nm showed more proliferation and adhesion whereas cells grown on tubes with 100 nm diameter display enhanced differentiation and reduced cell proliferation. Smaller diameter vertically aligned nanotubes adsorb more proteins due to greater surface area, thus promoting cell proliferation and attachment. In contrast, cells grown on larger diameter tubes must extend cell filopodia over larger distances across the lumen to attach to the protein adsorbed on the top surface of the tube. This leads to greater strain on the cell with effects on cell mechano-transduction thereby enhancing osteogenic differentiation in the process [163]. This emphasizes the dichotomy between cell differentiation and cell proliferation, with osteoblasts requiring signals from the implant surface to cease proliferation and start differentiation and subsequent bone deposition and mineralization. The exact dimensions of nanoscale titania surfaces most conducive to osteoblast differentiation is unclear with studies reporting nanotube diameters ranging from 15 to 100 nm and grain size for nanoscale surfaces ranging from 32 to 56 nm [42,163,164]. Such variation in ideal nanotube diameters likely stem from other variables that are not often characterized and compared between studies such as the composition of the scaffold and the degree of variations in nanotube height.

More recently, attention has been focused on combining titania nanotube coatings with underlying microstructured surfaces to enhance osteogenesis. Addition of titania nanotubes to micro-structured titanium further enhances osteoblast differentiation and collagen expression, increasing ALP activity and matrix bone matrix mineralization compared to plain microstructured scaffolds [165]. However, nanotextured surfaces without underlying microstructure show poor osteointegration. When purely nanostructured surfaces are implanted into rat femurs there was an initial period of bony ingrowth followed by a general decline in implant fixation strength that coincided with the gradual resumption of walking after surgery. Despite the bony ingrowth into the nanoarchitecture, the implant-bone interface was too weak and was disrupted by the gross motion of the rat. However, when the same implant incorporated underlying microstructure in addition to nanoscale architecture there was further improvement in implant fixation strength over standard micro-structured implants. This indicates that during initial healing, the micro-structured surface was able to enclose a greater volume of bony tissue in its grooves and depressions allowing for stronger immobilization and anchorage, thus allowing more time for further bone interdigitation into the nano-scale pores. In such situations the overlying nanoscale topography adds to the fixation strength of the underlying microstructured surface [166].

### 3.5. Growth Factors

During osteogenesis various growth factors are secreted by osteoprogenitor cells and osteoblasts to recruit mesenchymal cells and induce osteoblastic-lineage differentiation [29]. Osteogenic growth factors such as Fibroblast Growth Factor 2 (FGF2), TGF-β2 and BMP2 have been incorporated into to metallic implants as biologic coatings to improve its osteoinductivity [167,168,169,170]. TGF-β2 is a chemotactic factor that also promotes proliferation of osteoprogenitor cells and osteoblasts. FGF2 is a mitogenic factor for osteoblasts and mesenchymal cells secreted by osteoblasts, macrophages, osteoblasts and chondrocytes [171]. BMP2 is secreted by osteoblasts and osteoprogenitors cells to promote osteoblastic differentiation of mesenchymal stem cells [171]. Out of these growth factors BMP2 is the most commonly used growth factor used to improve osteointegration of metallic implants. It is upregulated during the first 3 weeks of osteogenesis [29]. BMP2 and BMP7 are approved by the United States food and drug administration (FDA) for treatment of fractures [172]. However, in order to achieve optimal results the growth factor must be delivered in a sustained fashion that emulates the natural release profile of BMP2 *in vivo*. Bolus delivery of BMP2 is inferior to sustained release of the growth factor in inducing new bone formation in extra-skeletal sites [173]. Bolus delivery of BMP2 likely leads to supra-physiological levels of the growth factor that can lead to unwanted ectopic ossifications, osteolysis and increased risk of tumorgenesis [174]. In the following paragraphs, the various means by which BMP2 can be incorporated into the coating of metallic implants will be discussed. Studies that mainly look at sustained delivery of BMP2 without further immobilization of the growth factor to metallic substrate will not be covered as they are mainly aimed at improving bone regeneration in general and not specifically aimed at implant osteointegration.

Various techniques are available to incorporate BMP2 into metallic implants (Table 1). A simple method is direct adsorption whereby the growth factor is adsorbed to the implant surface through non-covalent interaction. However, the main disadvantage of direct adsorption is its low growth factor retention time and inconsistent release profile, usually with significant burst release characteristics [175,176]. This increases the concentration of the growth factor needed to achieve the desired outcome and the chance of toxicity associated with supra-physiological drug levels. Another technique is to combine BMP2 to calcium phosphate coatings. The osteoinductive BMP2 combines with the osteoconductive calcium phosphate to deliver a multi-functional orthopaedic coating that is more effective than plain calcium phosphate coatings [170,177]. The porosity of the calcium phosphate is a critical factor that affects the osteoinductivity of BMP2-containing calcium phosphate coatings. In rat models of osteoinduction, bone formation is maximal when the pore size of BMP2-containing HA scaffolds is within 300–400 μm, this effect is diminished when pore size deviates from this value [178,179]. The pore size of calcium phosphate also affects the mode of ossification in response to BMP2. HA scaffolds with 300–400 μm pores display predominantly direct ossification with no preceding chondral stage while scaffolds with 90–100 μm pores first promote cartilage formation followed by enchondral ossification [180]. This likely relates to the reduced vascular infiltration owing to reduce pore sizes leading to reduced oxygen levels. 

**Table 1:** Various BMP2 (bone morphogenetic protein 2) sustained released mechanisms that can be engineered into metallic implants to promote osteoinduction. HA, hydroxyapatite; PEM, poly-electrolyte membranes; ECM, extracellular matrix molecules.

Study	BMP2 Sustained Delivery Mechanism	Category
Vehof *et al.* 2001 [170]	calcium phosphate loaded	Calcium phosphates
Ono *et al.* 1995 [177]	Calcium phosphate loaded	
Tsuruga *et al.* 1997 [178]	Calcium phosphate loaded	
Kuboki *et al.* 2001 [180]	Calcium phosphate loaded	
Liu *et al.* 2005 [181]	Co-precipitated calcium phosphate	
Kim *et al.* 2008 [182]	Poly(d,l-lactide-*co*-glycolide) (PLGA)/nanohydroxyapatite particles	
He *et al.* 2012 [70]	Calcium phosphate/collagen	ECM and chitosan
Bae *et al.* 2012 [183]	HA/chondroitin sulfate	
Schützenberger *et al.* 2012 [184]	Collagen sponge	
Geiger *et al.* 2003 [185]	Collagen sponge	
Dawson *et al.* 2009 [186]	Collagen sponge	
Abarrategi *et al.* 2008 and 2009 [187,188]	Chitosan film	
Yang *et al.* 2012 [189]	Heparin-conjugated fibrin	Heparin conjugation
Ishibe *et al.* 2009 [190]	Heparin/apatite	
Macdonald *et al.* 2011 [191]	Poly(β-aminoester)/chondroitin sulfate PEM	Polyelectrolyte membrane
Hu *et al.* 2012 [192]	Gelatin/chitosan PEM	
Shah *et al.* 2011 [193]	Poly(β-amino ester)/polyanion PEM	
Jiang *et al.* 2012 [194]	Hyaluronic acid/cationic liposome-DNA complex PEM (non-viral transfection)	non-viral based transfection with BMP2 gene
Hu *et al.* 2009 [195]	Chitosan (Chi) and plasmid DNA complex PEM (viral transfection)	
Qiao *et al.* 2013 [196]	PLGA microspheres containing BMP2 cDNA plasmid (viral transfection)	
Hu *et al.* 2012 [197]	TiO_2_ nanotubes	Titanium nanotubes
Lai *et al.* 2011 [198]	TiO_2_ nanotubes	

More recently there has been a trend to combining BMP2 and calcium phosphate with ECM molecules such as collagen and biodegradable polymers into one coating for implants. These new modes of growth factor delivery lengthen the release of BMP and showed promising results in osteoinduction in various animal models [70,182,183]. Osteoblasts grown on such surfaces also display enhanced proliferation and differentiation [183]. The release profile of BMP2 can also be improved by incorporation of BMP2 into the 3D lattice structure of HA by co-precipitation of BMP2 with HA. The BMP2 is gradually released into the cellular environment as HA is degraded extending the release time of the growth factor and improving scaffold osteoinductivity [181]. Heparin is another molecule that can be added to BMP2 loaded surfaces to improve the effectiveness of BMP2 delivery from the implant. BMP2 contains heparin binding sites at its basic-*N* terminal domain [199]. Binding of heparin to BMP2 protects it from degradation and bolsters osteoblast differentiation [200]. Addition of heparin to the coating surface maximizes the amount of immobilized BMP2 as well as effectively extending growth factor retention time [189,201]. Heparin-conjugated BMP2-containing scaffolds have been implanted in both skeletal and extra-skeletal sites in animals improving scaffold osteoinduction and osteointegration of metallic implants [190,201]. 

**Figure 5:**
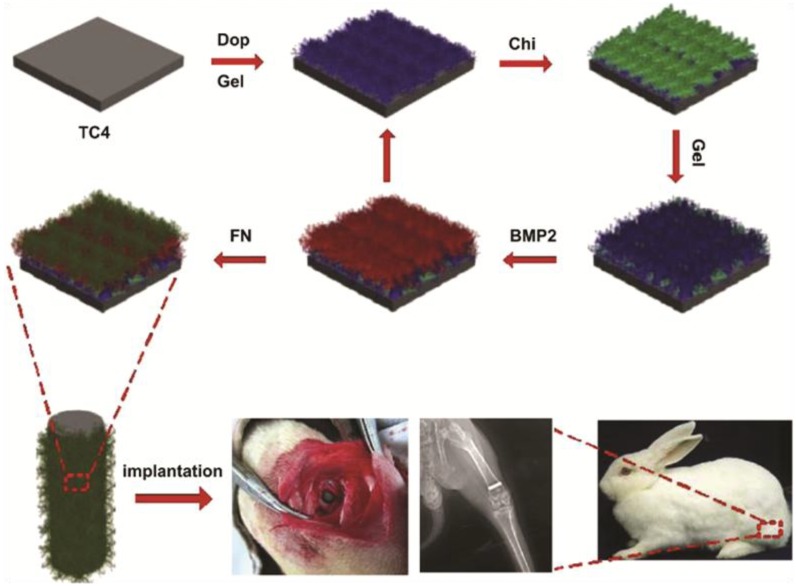
Schematic diagram showing production process of PEM consisting of layers of gelatin (Gel), chitosan (Chi), BMP2 and fibronectin (FN). Titanium alloy Ti6Al4V surfaces (TC4) is first coated with gelatin through dopamine (Dop) conjugation. This is followed by deposition of chitosan, BMP2 and fibronectin layers. Titanium rods coated with these PEM promote osteointegration when inserted into the femur of rabbits. Bottom row images show PEM coated titanium rods in the rabbit femur 4 weeks after implantation. PEM is a versatile and efficient means of complexing a range of biomolecules to metal surfaces. Image adapted from [192] with permission from Elsevier, Copyright 2014.

Incorporating BMP2 and other biological molecules to metallic implants often requires high processing temperatures under physiologically detrimental conditions leading to loss of biological activity of the complexed molecule. These technical hurdles can be overcome by development of layer-by-layer production of poly-electrolyte membranes (PEM). PEM consist of alternative layers of cations and anions that are deposited sequentially during production. The layers self-assemble due to the electrostatic attraction between the cationic and anionic layers. Various positively and negatively charged biomolecules, including BMP2, can be incorporated into the layers of PEM (Figure 5). PEM can be formed at room temperature under physiological conditions that do not lead to loss of bioactivity of incorporated molecules. It is a versatile and efficient means to controlling the physico-chemical properties of the coating surface [202]. Metallic implants with BMP2 containing PEM retain bioactivity for 1 year in storage and show sustained release of growth factor *in vitro* [203]. These membranes promote osteoblast differentiation *in vitro* and ectopic bone formation *in vivo* when implanted in extra-skeletal sites [191]. Polyelectrolyte membranes provide a means of combining BMP2 with multiple ECM molecules to produce multi-modal coatings for metallic orthopaedic implants. Titanium implants coated with PEM consisting of BMP2, fibronectin, chitosan and gelatin promote osteogenic-lineage differentiation of MSCs *in vitro* and increased bone formation when implanted into bone *in vivo* [192]. PEM technology also allows for co-administration of multiple growth factors with different release profiles. PEM films containing BMP2 and angiogenic factor VEGF are able to simultaneously release BMP2 over 2 weeks and VEGF over 8 days. The addition of VEGF to the PEM-BMP2 enhances osteoinduction *in vivo* by promoting vascular penetration of the scaffold with increased delivery of osteoprogenitors to the bulk of the scaffold leading to greater bone deposition [193,204]. 

Other recent studies have attempted to load implants with BMP2 DNA plasmids that are able to transfect osteocytes resulting in sustained secretion of BMP2 by transfected cells [194,195,196]. Titanium implants coated with BMP2 plasmids promote osteogenic differentiation of both osteoblasts and MSCs *in vitro* compared to non-coated titanium [194,195]. In addition to titanium, BMP2 plasmids can also be added to polyethylenimine (PEI) or calcium phosphates [195,196]. To further enhance the osteoinductivity, some researchers have managed to pre-transfect osteoblasts with BMP2 and angiogenic factor VEGF before they are seeded into CaPO_4_ scaffolds and implanted intramuscularly *in vivo* leading to enhanced vascularized bone formation [205]. However more research is required on the biosafety of BMP2 plasmid before it can be applied to humans. 

Apart from calcium phosphates and PEMs, other BMP2 carriers that are studied include ECM molecules, chitosan and titanium nanotubes. ECM contains many binding sites for BMP2. BMP2 containing ECM constructs show more sustained release of the growth factor compared to other substrates such as calcium phosphates [206]. Various ECM molecules are used as carriers of BMP2 such as collagen and fibrin [184,185,186,201]. Collagen scaffolds coated with BMP2 enhanced bone regeneration [185]. However, studies using collagen-coated metal implants as a means to carry BMP2 failed to show any benefit in osteoinduction over plain collagen-coated implants [157]. The difference in results may be due to technical parameters associated with coating metal surfaces with collagen. Chitosan, a polysaccharide extracted from crustaceans has also attracted attention as a possible carrier of BMP2. Chitosan film-based BMP2 delivery constructs are able to promote osteogenesis both *in vitro and in vivo* in animal ectopic ossification models [187,188]. More recently, with advances in nanotechnology, titania nanotubes are also employed as reservoirs of BMP2. Titanium oxide nanotubes loaded with BMP2 were covered by multilayered coating consisting of alternating chitosan/gelatin layers to allow for sustained release of BMP2 [197]. These constructs induced osteogenic differentiation of MSC *in vitro*. 

### 3.6. Bisphosphonates and Strontium

As the average life expectancy continues to soar in western societies the number of patients diagnosed with osteoporosis is expected to increase. Osteoporosis is a disease characterized by progressive loss of bone density and strength and is common in the elderly population. Osteoporosis impairs bone remodelling and healing after joint arthroplasties and fracture fixation reducing both bone-implant contact and peri-implant bone formation [207,208,209,210,211]. The quality of bone surrounding the implant can be improved with systemic bisphosphonate therapy [208]. Bisphosphonates act principally through inhibition of osteoclast induced bone resorption thus promoting net bone deposition. However, high doses of bisphosphonate are associated with gastrointestinal upsets, osteonecrosis of the jaw and increased fracture risk [212]. The systemic toxicity of bisphosphonates can be minimized by immobilization on orthopaedic implant surfaces. The immobilized bisphosphonates rarely diffuse far from the implant surface, minimizing the amount of drug entering the circulation and localizing its effect at the implant-bone interface [213]. Bisphosphonates can either be attached to implants through an interposing layer of calcium phosphate or fibrinogen [3,212,214,215,216,217]. The efficacy of different bisphosphonates in promoting implant osteointegration also varies. Zoledronic acid-containing titanium implants are more effective than Ibandronate or Parmidronate implants in improving peri-implant bone density, bone microarchitecture and strength of bone-implant bond in osteoporotic rats [212]. The effects of bisphosphonates can be further enhanced by co-immobilization of implants with growth factors such as basic fibroblast growth factor (bFGF) leading to improved bone-implant integration [214]. The anti-osteoclastic effects of the bisphosphonate combines favourably with the osteogenic differentiating effect of bFGF to promote bone remodelling. Apart from bisphosphonates, other coatings that show promise in promoting osteointegration of implants in osteoporotic bone include collagen, HA and adiponectin [218,219].

Bisphosphonate-loaded implants can also improve osteointegration in non-osteoporotic healthy bone. Titanium implants with parmidronate improved bone-implant bond in various animal models [3,216,220,221]. Bisphosphonate-containing titanium implants have also been tested in human patients with fibrinogen-coated titanium dental implants loaded with ibandronate and parmidronate improving implant osteointegration at six months after surgery [217]. Another anti-osteoporotic drug that is effective as an orthopaedic implant coating is strontium (Sr). Sr promotes osteoclast apoptosis through activating calcium sensing receptor (CaR), phospholipase C and NF-κB and osteoprogenitor proliferation and differentiation by upregulating Akt and PGE_2_ and the Wnt/cantenin pathway [4,222]. Like bisphosphonates, high systemic doses of strontium can lead to side effects such as osteomalacia [223,224]. Localized delivery of Sr through an implant based carrier system minimizes systemic toxicity while focusing activity to sites of bone-implant contact. Titanium implants containing strontium increase peri-prosthetic bone formation *in vivo* [225]. To extend the ion release time, Sr can be incorporated into the 3D lattice structure of titanium oxide layer on titanium implants through hydrothermal treatment. The SrTiO_3_ layer releases Sr in a sustained fashion and promotes osteoblast differentiation *in vitro* and bone implant apposition *in vivo* [226]. Titanium nanotubes are another means for delivery of Sr in a sustained fashion that can stimulate osteoblastic differentiation of MSCs [227]. Sr can also be combined with ECM molecules such as collagen to form composite coatings that draw on multiple molecular pathways to drive osteointegration [4].

## 4. Anti-Infection Coatings

Infection is a main cause of implant loosening after joint arthroplasty. In some cases this necessitates removal of the original prosthesis followed by delayed revision procedure to re-implant a new prosthesis back into the bone. In such cases, the patient needs to endure periods of immobility and accept higher chances of reinfection and loosening associated with the revision procedure. Much research has focused on developing orthopaedic coatings with anti-infective properties. However in order to create bactericidal surfaces, the mechanism of bacterial colonization of metallic surfaces and the various factors that affect this process must be first elucidated. The environment surrounding newly implanted orthopaedic prosthesis predisposes it to infection. Upon implantation, the metallic surface of the prosthesis attracts protein adsorption, such as fibronectin, which facilitates bacterial adhesion [228]. A foreign body response ensues, blunting the host immune system to combat bacteria. Under these conditions the infecting bacteria undergo layered proliferation and secrete a polysaccharide-based matrix to create a bacteria-matrix complex, known as a biofilm, that protects the bacteria from host immune defenses and anti-microbials [229,230,231]. Overtime some biofilms can slough off the implant and seed into surrounding regions, thus expanding the infectious field [229]. Given the difficulty associated with removing established biofilms, much attention has focused on creating implant coatings that kill bacteria in the early stages of adhesion, thereby preventing biofilm formation. To begin this discussion, the underlying principles of designing anti-infective coatings will be first discussed with special emphasis on the response of bacteria to different surface features. This is followed by an outline of the different types of anti-bacterial coatings that are being developed.

### 4.1. Bacterial Response to Surface Cues

The complex interaction between the host defense and the invading bacteria during prosthetic infections can be briefly summed up by the “race to the surface” theory [232]. This theory states that the fate of the implant, in the event of a bacterial infection, depends on the relative speed that bacteria and the osteogenic cells attach to the implant surface. If osteoblasts populate the implant surface before bacteria, the bacteria will die off and no infection ensues. However if bacteria colonize the implant before arrival of osteogenic cells prosthetic infection inevitably follows [232]. Therefore surface coatings that preferentially accelerate osteointegration also indirectly reduce the risk of bacterial infection. However, in designing implant coatings, one is often faced with dilemma that bacteria and host cells possess a very similar repertoire of adhesive mechanisms and respond to similar cues. As a result, metallic surfaces that promote osteointegration are also predisposed to bacterial adhesion. This is best illustrated by the response of bacteria to various implant surface features. Like osteoblasts, bacteria prefer surfaces with higher surface energy (hydrophilic), roughness and nanoscale architecture [233,234,235,236,237,238,239,240,241]. Although most bacteria have hydrophobic surfaces they preferentially bind to hydrophilic substrates as these surfaces are more likely to attract protein and natural apatite deposition [233,240,241]. Most studies on roughness and bacteria colonization concur that bacteria prefer rough substrates with a rise in adhesion when roughness Ra values exceeds a threshold of 0.2 μm [241]. However, some studies dispute this finding showing no consistent relationship between these two parameters [234,235,236]. This conundrum likely reflects differences in the shape of the microarchitecture. Surfaces may have the same roughness Ra value; however this does not account for different patterns in surface architecture or feature shapes. The importance of the shape of surface features is best demonstrated by one study which showed reduced *Staphylcoccus aureus (S. aureus)* adhesion on poly(dimethyl siloxane) elastomer (PDMSe) substrates with microtopography consisting of ribs arranged in a diamond like array like the surface of a fast moving shark compared to smooth surface substrates [242]. More recently bacterial adhesion on nanostructured metallic surfaces has been examined [239]. *S. aureus*, *Escherichia coli (E. coli)* and *Pseudomonas aeruginosa* (*P. aeruginosa*) show enhanced adhesion and biofilm production when cultured on nanoscale titanium films with 100–200 nm scale undulations with 10–15 μm spacing [239]. Nanotopography is more influential over bacterial behaviour compared to other surface features such as surface energy and surface charge [239]. Given the similar affinity to various surface cues more research needs to be focused on developing implant coatings that are able to exploit subtle differences in bacterial and cell response to surface topography.

### 4.2. Silver Coating

Various anti-infective agents can be added to the surface of orthopaedic implants to actively kill bacteria and prevent infection. Silver (Ag) is a commonly used agent in various anti-infective applications. Silver disrupts bacterial membranes and binds to bacterial DNA and to the sulfhydryl groups of metabolic enzymes in the bacterial electron transport chain, thus inactivating bacterial replication and key metabolic processes [243]. Silver-coated substrates prevent adhesion of *S. aureus* and *Staphylcoccus epidermidis in vitro* [244]. Silver coatings on megaprosthesis and fracture fixation pins reduce the rate of adhesion and infection by *S. aureus in vivo* [245,246]. Ag-coated fracture external fixation pins have also been examined in human studies, however these studies fail to demonstrate any advantage in reduction of pin site infections when silver-coated pins are used [247,248]. This may be related to the propensity of Ag to be released from the implant which can depend on the method used to immobilize Ag on the implant. Like other growth factors, Ag must be administered in a sustained fashion to minimize side effects and maximize its anti-microbial activities. High Ag levels associated with burst release is toxic to osteogenic cells [249,250,251]. Various carriers of Ag have been developed. Ag can be loaded onto calcium phosphate coatings to impart anti-microbial properties to metallic substrates. HA nanocrystals loaded with Ag show anti-microbial activity against *S. aureus* and *E. coli in vitro* [252]. Similar results are reported by others *in vitro* [249,253,254,255]. In *in vivo* studies, titanium implants thermal sprayed with HA-containing Ag, reduced methicillin resistant *S. aureus* (MRSA) colonization and adhesion when implanted subcutaneously into rats [11]. Other sustained delivery mechanisms of Ag include polyamide, titanium nanotubes, anti-abrasive ceramics and polyelectrolyte membranes [250,256,257]. Titanium nanotubes loaded with Ag particles are able to provide anti-bacterial activity against *S. aureus* for 30 days [250]. Polyelectrolyte membranes consisting of heparin, chitosan and Ag nanoparticles exhibited anti-bacterial activity against *E. coli in vitro* [257]. Ag can also be incorporated into anti-abrasion ceramics such as titanium nitride (TiN) and titanium carbonitride (TiCN) [258,259,260]. Both TiN and TiCN have been used as coatings for hip replacements and impart a low friction coating to orthopaedic implants reducing fretting and debris particle formation [261,262,263,264]. Addition of Ag to the ceramic film enhanced its antibacterial activity [258,259,260]. However, as the Ag content increased there was also a concomitant reduction in corrosion and wear resistance [259,265]. One study reported an optimal Ag density of 1 × 10^18^ ions/cm^2^ which represented a balance between anti-bacterial activity and corrosion resistance [265]. However, more studies are needed to verify the efficacy of Ag coatings on orthopaedic devices *in vivo*. Attention must also be focused on examining the mechanical properties of Ag coatings on orthopaedic implants given the high loading conditions of joint prosthesis *in vivo*.

### 4.3. Nitric Oxide

Nitric oxide (NO) is bactericidal towards both gram positive and negative bacteria and prevents bacterial adhesion [266,267]. As a strong oxidant, exposure can lead to oxidation of diverse membrane and cytoplasmic proteins. NO reacts with superoxide produced by macrophages to form peroxynitrite. Peroxynitrite damages bacterial membranes through peroxidation. This chemical also crosses the bacteria membrane to oxidize bacterial DNA, damaging its strands in the process [268]. NO is very unstable and is difficult to immobilize resulting in the use of NO donors such as diazeniumdiolates and nitrosothiols to produce coatings that release NO for anti-microbial activity [269,270]. Diazeniumdiolate has been incorporated into a silicone-based sol-gel derived film and implanted into subcutaneous pockets in rats that were infected with *S. aureus*. The NO-containing implants successfully reduced the rate of infection with *S. aureus* [12]. 

### 4.4. Chitosan

Chitosan is a polysaccharide derived from crustaceans (animals with hard exoskeletons) that has found use as a biocompatible scaffold in a range of tissue engineering applications. Chitosan also displays anti-bacterial properties through positive charged amino groups on the chitosan backbone that bind to negatively charged bacterial membranes, inducing membrane leakage [271]. Chitosan has been incorporated into various polyelectrolyte membranes on metallic implants. PEM with incorporated chitosan, heparin and silver nanoparticles shows anti-bacterial activity against *E. coli* [257,272]. However, the anti-bacterial effects of chitosan are limited as the amino groups on chitosan only display weak positive charges [273]. Furthermore chitosan is poorly soluble in water with pH of greater than 6.5 and is very brittle at room temperature [274,275]. As a result, chitosan has been chemically modified to address each of these issues. The positive charge of chitosan can be enhanced by addition of extra cationic charged groups to its backbone leading to enhancement of bactericidal activity. Examples of these derivatives include acyl thiourea and chitosan-*N*-arginine (CS-*N*-Arg) [273,276]. The water solubility of chitosan can also be improved by addition of fumaric acid or quaternary ammonium groups to form *O*-fumaryl-chitosan and quaternized chitosan respectively [274,277]. The mechanical properties of chitosan can be strengthened by blending it with polyethylene glycol fumarate [275]. These modifications bolster the antibacterial effects of chitosan [273,274,275,276,278]. However, more studies are needed to examine the anti-bacterial effects of these chitosan derivatives when they are used as coatings on metallic substrates.

### 4.5. Titanium Oxide Photocatalysis

Titanium oxide attains antimicrobial properties after irradiation by UV light. Under UV irradiation, titanium oxide reacts with the atmosphere and water to form superoxide and hydroxyl ions. These ions react with bacterial membranes causing oxidative damage, leading to derangement of bacterial proteins that rely on membrane integrity to function normally [279,280,281]. This process is known as photocatalysis. Thin TiO_2_ films show anti-bacterial activity against *E. coli* after UV irradiation [282]. Daily irradiation of TiO_2_ pins with UV light reduced the amount of MRSA colonization when they were inserted into rabbit femurs [13]. More recently it was discovered that addition of Ag cations to the titanium oxide can bolster photocatalysis, improving the efficacy of its anti-microbial activity. The Ag nanoparticles enhance the antibacterial activity of TiO_2_ by increasing UV ray absorption rather than through Ag ion elution [283,284]. Given the potentially harmful effects associated with UV light exposure, other groups have modified TiO_2_ with carbon (C). Carbon-containing titania is anti-microbial against *S. aureus*, *Shigella flexneri* and *Acinetobacter baumannii* upon illumination with visible light [280]. However the requirement for implant exposure for UV/light irradiation limits the application of these devices in clinical situations.

### 4.6. Antibiotic Elution

Antibiotics have traditionally been incorporated into polymethyl methacrylate (PMMA) cement during cemented joint arthroplasties. However, antibiotic-loaded PMMA suffers from several main disadvantages. Firstly, PMMA cement loaded with antibiotics shows rapid, unreliable and incomplete drug release profiles. Only 20% of gentamicin is released from PMMA cement for the duration of hip implant function [285]; Secondly, antibiotics can affect the mechanical properties of the PMMA cement accelerating implant loosening. Vancomycin reduces the bending and fatigue strength of PMMA cement [286,287]; Thirdly, the heat energy released during setting of PMMA cement during arthroplasties limits the choice of antibiotics to those that are heat stable. As a result, much research has focused on developing new means of immobilising antibiotics to implants. Biodegradable polymers, calcium phosphates and titanium nanotubes are investigated as antibiotic-eluting coatings for orthopaedic implants. 

Biodegradable polymers provide a reliable means to deliver antibiotics in a sustained and controllable fashion. Polymer microspheres based on polyparadioxanone (PPD), polyglycolic acid (PGA), or polylactic acid (PLA) can be successfully loaded with antibiotics and further immobilized to metallic substrates [288,289,290]. Unlike PMMA cements, polymer microspheres are capable of completely releasing all antibiotics in a sustained fashion thus minimizing any local or systemic toxicity associated with high fluctuating antibiotic concentrations. Gentamicin-loaded poly-l-lactide (PLLA) coatings can release 80% of the gentamicin in six weeks thus providing sustained and near complete elution of antibiotics [291]. Gentamicin-loaded PDLLA (poly(d,l-lactide)-coated titanium implants reduced the risk of osteomyelitis by 90% when implanted into rat tibial medullary canals inoculated with *S. aureus* [14,292]. In addition to antibiotics, antiseptics can also be immobilized to polymer coatings on orthopaedic implants. The antiseptics Octenidin and Irgasan reduced the rate of osteomyelitis when loaded onto PLLA-coated titanium plates and inserted into rabbit tibias infected with *S. aureus*. These antiseptics are just as effective as antibiotics in reducing bacterial infections [291]. The release profile of antimicrobials from polymer carriers can be fine-tuned by altering the polymer/solvent/drug ratio. One study found by increasing the PDLLA and reducing the gentamicin level the release of gentamicin from PDLLA implant coatings can be prolonged [293]. The main disadvantage of antibiotic-eluting polymer coatings is their lack of biologically active surfaces. This can potentially be compensated by combination with other biological coatings that promote osteointegration.

Both calcium phosphate and titanium nanotubes have been investigated as possible carriers of antibiotics. Stainless steel k-wires coated with gentamicin loaded HA reduced the rate of infections when they are inserted into rabbit tibia, previously inoculated with *S. aureus* [294]. Calcium phosphate-based antibiotic delivery systems show greater anti-microbial activity compared to bone cement-based carriers likely due to more complete elution of antibiotics. Vancomycin-coated HA beads are more effective than Vancomycin-coated PMMA beads in reducing the rate of osteomyelitis when inserted into infected tibias in rabbits [295]. In addition to delivering antibiotics, calcium phosphate coatings can also deliver antiseptic agents thus reducing the risk of antibiotic overuse and resistance. HA-coated stainless steel pins loaded with chlorhexidine reduce the rate of *S. aureus* infection by 83.3% when implanted into infected goat tibias [15]. Titanium nanotubes have received much attention as a carrier of various drugs. Titanium nanotubes can be produced by anodizing titanium surfaces to generate nano-tubular surface structures. Titanium nanotubes are capable of sequestering antibiotics and delivering them in a sustained, localized fashion. Titania nanotubes loaded with gentamicin are effective in reducing the number of colony forming units *of S. epidermidis* on its surface. The antibiotic was fully eluted over 160 min with no impact on the osteoconductive and osteoinductive properties of titania nanotubes [296]. The rate of antibiotic elution can be controlled by varying the diameter of the nanotubes. Titania nanotubes with diameters of 160 to 200 nm released antibiotics at a slower rate compared to smaller nanotubes with diameter of 80–120 nm and were more effective than the later in treating *S. epidermidis* infection *in vitro* [297]. The elution time of antibiotic from titania nanotubes can be further extended by immersing nanotubular metals in physiological solutions containing antibiotics that facilitates co-precipitation of natural apatite with the antibiotics onto the metal surface. This extended the elution time of penicillin based antibiotics to over 3 weeks [17].

### 4.7. Antimicrobial Tethering

Antibiotic-eluting implant coatings suffer from several disadvantages. Firstly antibiotic-eluting coatings can only release antibiotics at therapeutic concentrations for a limited period of time. As the antibiotic is depleted the drug concentration surrounding the implant drops to sub-therapeutic levels enabling bacteria that have managed to temporarily evade treatment to re-colonize the implant. Secondly, low antibiotic concentrations impose a selectional pressure on the remaining bacteria driving the development of resistance to antibiotics in bacteria [229]. In fact, culture of PMMA beads loaded with gentamicin extracted during revision procedures on patients with infected orthopaedic prosthesis show growth of gentamicin resistant bacteria due to sub-therapeutic gentamicin content [298]. Where antibiotic mechanism permits, shortcomings of elution can be solved by tethering antibiotics to the implant surface. Tethered anti-microbials will not detach from the implant providing a permanent anti-bacterial surface that lasts for the life span of the implant. Various antibiotics with membrane disruptive mechanism and antiseptics have been immobilized to metallic implants. For example, Vancomycin, which acts on the bacterial cell wall synthesis, covalently linked to titanium implants prevents *S. aureus* colonization and biofilm formation by *S. epidermidis in vitro* [299,300]. This antimicrobial activity is preserved even after 11 months of immersion in PBS [301]. Similar effects have been shown in animal studies. Titanium rods with immobilized Vancomycin reduce *S. aureus* colonization and biofilm formation when implanted into infected femoral medullary canals in rats [16]. However, tethering is not applicable to antibiotics that target cytoplasmic proteins as they need to diffuse from the implant to cross the bacterial membrane. 

With increasing use of antibiotics in both medicine and industry the incidence of antibiotic resistance is rising rapidly, placing greater burden on health systems and driving the search for new anti-microbial agents. One type of anti-infective agent that has received renewed attention is the anti-microbial peptide. Anti-microbial peptides are sequences of 40–50 amino acid residues that are synthesized by mammals, amphibians and plants to combat infection. They are generally hydrophobic and cationic containing an abundance of charged amino acids that form amphiphilic α helical structures suited to binding to the negatively charged cell membranes of bacteria. Anti-microbial peptides generally function by disrupting bacterial membranes [302]. Various anti-microbial peptides can be tethered to metallic implants to provide an effective anti-infective coating. Compared to antibiotic coatings, anti-microbial peptide coatings enjoy the advantage of heightened bacterial specificity with minimal toxicity to host cells. Anti-microbial peptides also reduce the usage of antibiotics thus reducing the risk of drug resistance. Titanium substrates immobilized with the antimicrobial peptide LL-37, showed bactericidal effects on *E. coli* [303]. Another antimicrobial peptide Magainin I immobilized to gold through a self-assembled thiol-containing monolayer showed anti-microbial activity against *Listeria ivanovii*, *Enterococcus faecalis* and *S. aureus* for six months *in vitro* [18,304]. However the main limitation of antibiotic and anti-microbial peptide tethering is a lack of antimicrobial impact on bacteria that are not in direct apposition to the implant. This is especially relevant in revision arthroplasties where the soft tissue surrounding the bone also contains biofilms, which can act as a separate source of infection. Future anti-infective coatings should combine both antimicrobial tethering and antibiotic-eluting mechanisms into one coating to provide close as well as distant defense against invading bacteria.

## 5. Conclusions and Future Directions

Advances in manufacturing, cell biology and material science have driven the development of new biological coatings for orthopaedic implants that aim to recapitulate the natural environment of growing bone. Coatings consisting of calcium phosphates, ECM peptides and immobilized growth factors exploit the natural cellular mechanisms underlying osteogenesis to promote osteointegration of the implant. The design of osteogenic coatings must also account for anti-infective requirements of orthopaedic devices. Metallic surfaces fashioned with Ag, NO-generating agents and antibiotics have all shown promise in a range of *in vitro* and *in vivo* studies in reducing both bacterial adhesion and viability. The next step in this field is to combine the various osteogenic and anti-infective coatings and draw on the advantages of each class of material to engineer composite structures that can reduce the risk of both aseptic and infective loosening in joint arthroplasties. However before this goal can be realized, certain barriers need to be overcome. Firstly, more study is required to explore differences between cell and bacterial response to various surfaces and materials. Such insight will aid in directing the design of scaffolds that are able to exploit these subtle differences in biology to selectively promote bone growth while retarding bacterial adhesion. Secondly, standardization is required for experiments on osteoinduction and osteoconduction. Due to differences in osteoinductive capacities between various animal species a consensus needs to be established in regards to the type of animal model that all studies should utilise to simplify inter-study comparisons and data interpretation. The type of animal model used should take into consideration differences between human and animal biology and whether findings in animal models regarding osteoinduction can be translated to human subjects. Establishing a uniform animal model of osteoinduction would also aid in reducing the variability that currently exists with critical experimental results such as ideal dimensions and compositions of scaffolds for bony ingrowth. 
